# Supplementary material for: Acceptance and compliance with micronutrient powder (MNP) among children aged 6–23 months in northern Nigeria
Source: PLOS Glob Public Health. 2022 Oct 17;2(10):e0000961. doi: 10.1371/journal.pgph.0000961 (PMC10022258; doi:10.1371/journal.pgph.0000961)
Supplement: S1 Dataset — (ZIP) [file pgph.0000961.s008.zip › S8_Dataset/Spot Checks.zip!/Spot Checks/08072015_A_GA_AD_2_SC.pdf]

SPOT CHECK RECORDING FORM

State: Adamawa _ LGA:_Ganye__________ Community: __Dadiri_

Name of caregiver (female):……..Zainab Saleh…….

Name of caregiver (male): ………………..……………….._

Sex of index child: female_ Age of index child (in months): 17 month________

Spot Check Information

1. **Date of MNP distribution to household** (circle option below based on LGA name above)**:**
   - **Kebbi:** BirninKebbi&Jega LGA: **June 16**
   - **Kebbi:** Danko-wasagu: **June 17**
   - **Adamawa:**Song, Ganye: **June 18**
   - **Adamawa:**Guyuk **June 19**
2. **Date of Spot Check Observation: ___08/07/2015_____**
3. **Number of sachets remaining**(Ask the caregiver to show you and count the specific number of sachets remaining)**:**

   55

4. **Number of sachets that should remainusing one MNP sachet per day** (Analyst only)**:**

   41

5. **Number of sachets remaining / number of sachets should be remaining**(Analyst only)**:**

   _55________ / ___41_____ = ____134_____ %

Probes

Question 1. You have **XX** MNP sachets remaining. Explain how easy or difficult it was to use the MNP product on a daily basis for your child 6 – 23 months? (Circle one answer)

**Very Difficult**	**Difficult**	**Easy**	**Very Easy**

Question 2. Describe any specific challenges that you faced that made the MNP difficult to use on a daily basis?

I have to use a razor blade to open the sachet.
Preparing kunu each day just to administer the MNP is that ok.
Can I still continue administering the MNP because my child is feeding better now

Question 3. Describe specific aspects of the MNP product that you or your child enjoyed that made it easier to use on a daily basis?

The taste was ok because my child did not reject it in the few days that I gave her

Question 4. What questions do you have about the MNP based on your experience using it?

Is diarrhoea a side effect of the MNP**?**
My child was vomiting breast milk after consuming MNP is that ok
Can I still continue administering the MNP? Because my child is feeling better now?

Question 5. What specific recommendations do you have that we can do as a program to make the MNP easier to use on a daily basis?

There should be no restrictions in the kind of food MNP will be added to
